# Congenital Heart Defects in Monochorionic Twins: A Systematic Review and Meta-Analysis

**DOI:** 10.3390/jcm8060902

**Published:** 2019-06-24

**Authors:** Manon Gijtenbeek, Maryam R. Shirzada, Arend D. J. Ten Harkel, Dick Oepkes, Monique C. Haak

**Affiliations:** 1 Division of Fetal Medicine, Department of Obstetrics, Leiden University Medical Center, PO Box 9600, NL-2300 RC Leiden, The Netherlands; m.r.shirzada@lumc.nl (M.R.S.); d.oepkes@lumc.nl (D.O.); m.c.haak@lumc.nl (M.C.H.); 2 Division of Pediatric Cardiology, Department of Pediatrics, Leiden University Medical Center, PO Box 9600, NL-2300 RC Leiden, The Netherlands; A.D.J.ten_Harkel@lumc.nl

**Keywords:** congenital heart defect, monochorionic twin pregnancy, prevalence, twin–twin transfusion syndrome, newborn

## Abstract

Monochorionic (MC) twins are at an increased risk of developing congenital heart defects (CHDs) compared to singletons and dichorionic twins. The development of acquired CHDs in this specific group of twins is associated with twin–twin transfusion syndrome (TTTS). We performed a systematic review and meta-analysis to provide an overview of the reported birth prevalence of CHDs in liveborn MC twins with and without TTTS. Twelve studies were included in this review. Compared to the reference population, MC twins were 6.3 times more likely to be born with a CHD (59.3 per 1000 liveborn twins; relative risk (RR) 6.3; 95% confidence interval (CI): 4.4–9.1), and TTTS twins had a 12-fold increased risk of having a CHD at birth (87.3 per 1000 live births; RR 12.4, 95% CI: 8.6–17.8). The increased incidence of CHDs can mainly be attributed to the risk of right ventricular outflow tract obstruction (35/1000 TTTS twin live births vs. 0.5/1000 singleton live births). We recommend an expert fetal echocardiogram in all MC twins, follow-up scans in the event of TTTS, and a postnatal cardiac evaluation in all TTTS survivors.

## 1. Introduction

Congenital heart defects (CHDs) represent the most common human birth defect, having a birth prevalence of 7–9 per 1000 singleton live births [1,2]. CHDs are more common in twin pregnancies with a reported prevalence of approximately 20 in 1000 live births. Monochorionic (MC) twins are at an even higher risk compared to dichorionic (DC) twins [2]. A systematic review and meta-analysis of four studies conducted in 2007 showed a 9-fold increase in CHD risk in MC twins [3] compared to singletons. 

The development of acquired CHDs in MC twins is associated with twin–twin transfusion syndrome (TTTS) [4]. TTTS complicates 10–15% of MC twin pregnancies and results from unbalanced blood flow from one twin (donor) to the other twin (recipient) via placental vascular anastomoses [5,6]. The birth prevalence of MC twins with a CHD may be influenced by the improved survival rates for MC twins over the last decade, especially for those treated for TTTS [7]. The literature has been significantly expanded and more up-to-date population prevalence rates have been published [1]. The aim of this systematic review and meta-analysis was to provide an updated overview of the reported birth prevalence of CHDs in liveborn MC twins with and without TTTS. 

## 2. Methods

### 2.1. Search Strategy

This systematic review was performed using the PRISMA methodology [8]. Relevant articles were identified using electronic databases (Pubmed, Embase, Web of Science, and Cochrane) on 17 January 2019, using search terms related to ‘monochorionic twins’ and ‘congenital heart defects’. The search was limited to original research papers with English abstracts. No time restriction for publication dates was used. All titles and abstracts were screened for study population (liveborn MC twins), type of CHD, and birth prevalence. Papers focusing on etiology, prenatal diagnosis, prognosis, or animal research were excluded. Two reviewers (M.G. and A.S.) screened titles and abstracts independently for relevance. If a title or abstract seemed relevant, full text was retrieved and assessed for inclusion. Articles were eligible if the number of liveborn MC twins affected by CHD could be determined from the published data, there was postnatal confirmation of the CHD, and chorionicity was determined. Selected articles were cross-referenced. Disagreement was resolved by consensus. 

### 2.2. Quality Assessment

Study quality and risk of bias was assessed by the two reviewers using the Hayden bias rating tool [9], as suggested by the Cochrane Collaboration. With this tool the risk of bias was assessed in six domains (study participation, study attrition, prognostic factor measurement, outcome measurement, study confounding, and statistical analysis and reporting). Each of the six potential bias domains was rated as having high, moderate, or low risk of bias. Low methodological quality was not an exclusion criterion. 

### 2.3. Data Extraction

Two reviewers (M.G. and A.S.) extracted the relevant information from the selected articles. The following study characteristics were extracted from the selected articles and tabulated: first author, year of publication, time period during which the study was performed, country, study design (retrospective or prospective), determination of chorionicity, number of live births, number of patients with CHD, birth prevalence of total CHDs, and prevalence of common CHD subtypes: right ventricular outflow tract obstruction (RVOTO), ventricular septal defect (VSD), atrial septal defect (ASD), coarctation of the aorta (CoA), aortic stenosis (AS), tetralogy of Fallot (TOF), and transposition of the great arteries (TGA).

### 2.4. Statistical Analysis

Statistical analyses were performed using MS excel for Windows (Microsoft Corporation, Redmond, Washington, DC, USA) and Review Manager 5.3 (Copenhagen: The Nordic Cochrane Center, The Cochrane Collaboration, 2014). Relative risks (RRs) and their 95% confidence intervals (CIs) were used as effect sizes for the meta-analysis of dichotomous data. Heterogeneity between studies was examined with the inconsistency square (I^2^) statistics, with between-study heterogeneity at I^2^ ≥ 50% and *p* ≥ 0.05 [10]. In case of heterogeneity a random effects model was used [11]. The population risk of CHDs was based on the study by Van der Linde et al. [1]. 

## 3. Results

**Figure 1:**
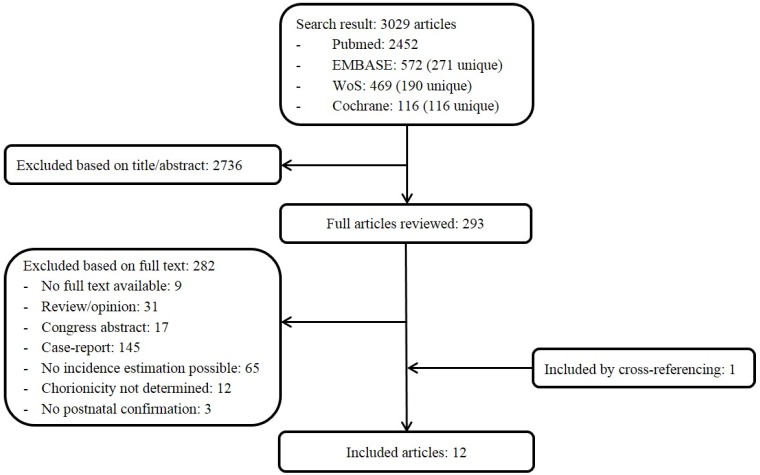
PRISMA diagram for study selection. WoS, Web of Science.

The systematic literature search yielded 3029 citations, of which 2736 were excluded by review of the title or abstract. Full manuscripts were retrieved for the remaining 293 studies and a total of 12 articles (*n* = 3136 liveborn twins) were included in the review (Figure 1, Table 1) [12,13,14,15,16,17,18,19,20,21,22,23]. Eight studies had a prospective design. Six studies included MC twin pregnancies complicated by TTTS only. Four studies only described the prevalence of RVOTO. There was some overlap between the cohorts of Lopriore et al. [18], Hack et al. [14], and Eschbach et al. [13]. Quality assessment is summarized in Table 2. To judge the overall risk of bias in each study, it is not recommended to use a summated score for the overall study quality [9]. In the included studies there was a low to moderate risk of bias in the majority of domains. Some studies did not report on their diagnostic tests; prognostic factor measurement was therefore rated as ‘high risk of bias’. There was a high risk of bias in the outcome measurement in the study by Hack et al. [14] since the authors only recorded the presence of congenital heart malformations without mentioning whether the method and setting of their investigations to diagnose a CHD was the same for all study participants.

**Figure 2:**
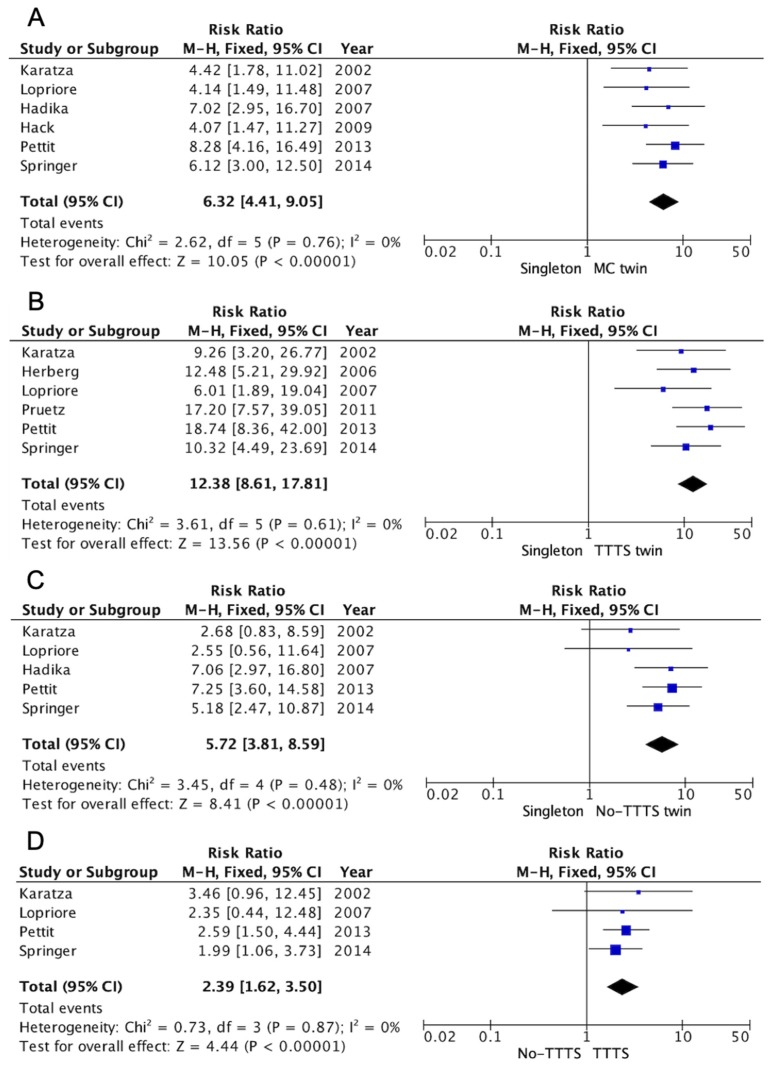
Risk of CHDs in MC twins with and without TTTS. (**A**) MC twins vs. singletons, (**B**) MC twins with TTTS vs. singletons, (**C**) MC twins without TTTS vs. singletons, (**D**) MC twins with TTTS vs. MC twins without TTTS. Risk ratios with 95% confidence intervals (CIs) were calculated by a fixed effect model. The pooled risk ratio is represented by a black diamond, where diamond width corresponds to 95% CI bounds.

In the study population of 3136 liveborn twins, 185 CHDs were identified. The prevalence of CHDs in MC twins was 59.3 per 1000 live births (95% CI: 50.5–69.4). In MC twins with and without TTTS, the prevalence of CHDs per 1000 live births was 87.3 (95% CI: 87.3–140.9) and 3.4 (95% CI: 44.2–64.5), respectively. Compared to the population prevalence of 9.1 per 1000 live births [1], MC twins were 6.3 times more likely to be born with a CHD than infants in the general population (RR 6.3; 95% CI: 4.4–9.1). TTTS twins were almost 2.5 times more likely to have a CHD than non-TTTS twins (RR 2.4; 95% CI: 1.6–3.5). Compared to singletons, TTTS twins had a 12-fold increased risk of having a CHD at birth (RR 12.4, 95% CI: 8.6–17.8) (Figure 2). 

Quintero’s classification to stage TTTS severity has been applied since 2000 [24]; studies investigating patient cohorts prior to 2000 therefore do not report Quintero stages. Hidaka et al. [16] describes one TTTS case (Quintero stage 2) where the donor appeared to have CoA after birth. Three of the studies report on the Quintero stage distribution in the study population. In the first study from 2007 [18], with a CHD prevalence of 5.4% in TTTS twins, the Quintero stage distribution was: 17% stage I, 37% stage II, 41% stage III, 4% stage IV. In the second study from 2011 [21], with a CHD prevalence of 15.5%, the Quintero stage distribution was: 10% stage I, 22% stage II, 50% stage III, 18% stage IV. In the third study from 2014 [23], with a CHD prevalence in TTTS twins of 8.9%, 30% of pregnancies were Quintero stage I, 40% stage II, 21% stage III, 1% stage IV, and 7% stage V. Eschbach et al. [13] found that 82% of RVOTO cases were staged as Quintero stage III or IV, compared to 43% of cases without RVOTO (*p* = 0.07).

The reported birth prevalence of the CHD subtypes in all MC twins (per 1000 live births) was: VSD, 25.9 (95% CI: 20.2–33.2); RVOTO, 22.3 (95% CI: 17.6–28.4); ASD, 13.6 (95% CI: 9.7–19.1); CoA, 2.1 (95% CI: 0.9–5.0); AS, 2.6 (95% CI: 1.2–5.6); TOF, 0.9 (95% CI: 0.2–3.1), and TGA, 0.9 (95% CI: 0.2–3.1). The prevalence of TOF and TGA was similar to the prevalence in singletons (both 0.3 per 1000 singleton live births). All other subtypes had a higher prevalence (*p* < 0.05). The type of CHD with the largest relative risk (RR 70; 95% CI: 27–179, *p* < 0.001) in TTTS twins was RVOTO (35/1000 vs. 0.5/1000 singleton live births). 

## 4. Discussion

With this systematic review and meta-analysis, we estimated the prevalence of CHD in MC twins to be 59 per 1000 live births, which is over 6 times higher as compared to singleton live births. In TTTS survivors the risk is even higher, with a 12-fold increased risk compared to singletons. The estimated prevalence in these neonates is 87 per 1000 live births. Therefore, we recommend an expert fetal echocardiogram in all MC twins at mid-gestation. In the event of TTTS, a second prenatal fetal echocardiogram around 30–32 weeks should be performed to rule out any acquired defects such as RVOTO, and a postnatal echocardiogram in all survivors may be considered.

The estimated prevalence rates and relative risks in this study are lower than those previously reported by Bahtiyar et al. [3]. There may be several explanations for this. First, the present study involves over 5 times the number of live birth MC twins, which enabled us to estimate the birth prevalence of CHD in MC twins with and without TTTS more precisely, and which possibly reduced the risk of selection bias. Second, we excluded stillbirths. The inclusion of stillborn fetuses would have elevated the prevalence of CHD. Finally, lower relative risks were calculated due to the use of the generally accepted population prevalence of CHDs of 9.1 per 1000 live births [1] instead of the lower rates from the cohorts of Wren et al. [25] or Ferenc et al. [26].

Twin birth rates have increased over the last decades due to the increasing maternal age and the extensive use of assisted reproductive technology (ART) [27,28]. ART increases not only the number of dizygotic but also the number of monozygotic twins. In MC twins, which are all monozygotic, the division of the fertilized ovum is hypothesized to be an influencing factor which could contribute to primary structural cardiac anomalies [29]. ART itself is also considered a risk factor for CHDs [30,31]. However, the increased incidence of acquired CHDs in MC twins has mostly been attributed to MC placentation and TTTS, indicating an influence of hemodynamic alterations on cardiac development. We found an increased risk of the most prevalent subtypes of CHDs (VSD, RVOTO, ASD, CoA, and AS) in MC twins compared with singletons, although this should be interpreted with caution due to the low numbers of some CHDs, particularly CoA and AS. However, this finding possibly supports the hypothesis of the influence of hemodynamic factors in the development of CHDs, which is furthermore supported by the fact that defects such as TOF, for which genetic influences are thought to be more important in development, are equally prevalent in MC twins and singletons. Previous studies suggest that more severe TTTS is associated with cardiac defects [4,32,33], possibly indicating an effect of a larger hemodynamic imbalance. This finding could not be supported by this meta-analysis since only a small number of studies report on the Quintero stage distribution [18,21,23], and in only one study the disease severity was analyzed in relation to CHD prevalence [13]. 

Fetoscopic laser surgery, as a curative treatment for TTTS, ensures cardiovascular improvement in affected twins [34,35,36] but does not prevent the occurrence of cardiac defects at birth in all cases, as shown by this study. Cardiac adaptation in TTTS mainly occurs in recipients [23,37]. Cardiac overload and hypervolemia in these twins may result in shear stress and ventricular hypertrophy, which can cause abnormal development of the cardiac valves through a cascade of events. Shear stress causes endothelial changes, and right ventricular hypertrophy and severe tricuspid valve regurgitation lead to diminished flow across the right ventricular outflow tract, which may impair growth and development of the right ventricular outflow tract. These processes can lead to RVOTO, which is found in approximately 3.5% of recipients (this study). It is suggested that since valve development is not completed at the beginning of the second trimester, fetuses who experience TTTS earlier in gestation are more frequently affected by RVOTO [13]. 

Less reported, but still clinically important, is the coexistence of CoA and TTTS, which seems to be more frequently seen in donors than in recipients [38]. The underlying mechanism leading to CoA is not fully understood. A proposed explanation is the reduced flow theory, which suggests that the narrowing of the aortic arch develops secondary to hemodynamic disturbances [39]. Decreased flow may occur as the result of decreased left-sided cardiac output of the donor twin in TTTS due to hypovolemia, or in the case of ventricular outflow tract obstruction [40].

Improved echocardiographic techniques are likely to substantially account for the increased detection rate of cardiac lesions. In the last decade there has been a shift towards a diagnosis before birth. In expert hands, prenatal detection rates of CHD in multiple pregnancies can be as high as 88% [41]. However, in the case of TTTS, the CHD detection rates are reported to be as low as 42.9% in recipient twins and 16.7% in donor twins [21]. Possible explanations for the low detection rates are the polyhydramnios in combination with the excessive movements of the recipient twin and the ‘stuck’ anhydramniotic donor, which both severely impair image acquisition and the detection of CHD. Therefore, next to the detection of possible acquired valvular pathology, follow-up fetal echocardiograms are warranted after TTTS treatment, when scanning conditions normalize, to rule out missed structural anomalies at earlier scans. An accurate diagnosis is critical in determining the requirement of immediate (postnatal) treatment, predicting the course of (surgical) repair, and for the counseling the parents about the prognosis. 

This study has certain limitations. There are only a few studies with a large sample size available. Comparisons of prevalence rates of all CHD subtypes between MC twins with and without TTTS and between MC twins and singletons are therefore limited. We found a high incidence of CHDs in MC twins, especially in the TTTS population, but it is possible that many milder forms of CHDs are present in twins without TTTS and in singletons that are missed or underdiagnosed, which could lead to an underestimation of the CHD prevalence in these infants. In this review, hospital-based studies were included which could have resulted in upwardly biased estimates of prevalence compared to national registries. Our data do not reflect the CHD prevalence at mid-gestation, since (selective) feticide cases and studies without postnatal follow-up were excluded. We do not think, however, that the inclusion of the (limited number of) feticide cases would have changed our results significantly. Despite these limitations, our results do suggest a significant burden of CHDs in MC twins that can have important neonatal implications. Future studies should determine whether there is still a need to perform postnatal echocardiography in all TTTS twins.

## 5. Conclusions

There is still a large burden of CHDs in MC twins with and without TTTS. We recommend an expert fetal echocardiogram in all MC twins, follow-up scans in the event of TTTS, and a postnatal cardiac evaluation in all TTTS survivors.

## Figures and Tables

**Table 1:** Article characteristics.

	Year	Author	Country	Time Period	Design	Chorionicity Determination	Study Population	Number of Liveborn Twins (*n* TTTS)	Number of CHDs
**1**	1996	Cincotta	UK	1994–1995	P	TTTS diagnosis	14 TTTS pregnancies	22	2/10 recipients RVOTO, donors 0
**2**	1998	Simpson	USA	1992–1997	P	Examination placenta postpartum	12 TTTS pregnancies	22	3/10 recipients RVOTO, donors 0
**3**	2001	Lougheed	Canada	1994–1998	R	TTTS diagnosis	73 TTTS pregnancies	146	6/73 recipients RVOTO, donors 0
**4**	2002	Karatza	UK	1997–2000	P	Examination placenta postpartum	136 MC twin pregnancies (47 TTTS)	226 (60)	9/226 MC twins, no-TTTS 4/166, TTTS 5/60
**5**	2006	Herberg	Germany	1995–1997	P	TTTS diagnosis, treated with FLS	73 TTTS pregnancies	89	10/89 TTTS twins
**6**	2007	Hidaka	Japan	2000–2006	P	Examination placenta postpartum	87 MC twin pregnancies (1 TTTS)	174 (2)	11/174 MC twins
**7**	2007	Lopriore	Netherlands	2002–2005	P	Examination placenta postpartum	101 MC twin pregnancies (46 TTTS)	161 (74)	6/161 MC twins, no-TTTS 2/87, TTTS 4/74
**8**	2009	Hack	Netherlands	2000–2007	R	First trimester ultrasound scan and/or examination placenta postpartum	98 MCMA twin pregnancies (6 TTTS)	164 (unknown)	7/164 MC twins
**9**	2011	Pruetz	USA	2009–2010	P	TTTS diagnosis, all treated with FLS	50 TTTS pregnancies	84	13/84 TTTS twins
**10**	2013	Pettit	USA	1996–2003	R	Examination placenta postpartum	482 MC twin pregnancies (48 TTTS)	926 (83)	69/926 MC twins, no-TTTS 55/843, TTTS 14/83
**11**	2014	Springer	Austria	2002–2012	R	First trimester scan, TTTS treated with FLS	381 MC twin pregnancies (70 TTTS)	754 (135)	39/754 MC twins, no-TTTS 27/619, TTTS 12/135
**12**	2016	Eschbach	Netherlands	2004–2015	P	TTTS diagnosis, majority treated with FLS	485 TTTS pregnancies	368 (368 recipients)	11/368 recipients RVOTO

P, prospective; R, retrospective; FLS, fetoscopic laser surgery; TTTS, twin–twin transfusion syndrome; CHDs, congenital heart defects; MC, monochorionic; RVOTO, right ventricular outflow tract obstruction.

**Table 2:** Quality scores based on the Hayden bias rating tool.

	Variable/Study	Study Participation	Study Attrition	Prognostic Factor Measurement	Outcome Measurement	Study Confounding	Statistical Analysis and Reporting
1	Cincotta	Moderate	Moderate	High	Moderate	Low	Low
2	Simpson	Low	Low	Low	Moderate	Low	Low
3	Lougheed	Moderate	Moderate	High	Low	Low	Moderate
4	Karatza	Low	Low	Low	Low	Low	Moderate
5	Herberg	Low	Low	Low	Low	Low	Low
6	Hidaka	Moderate	Low	Moderate	Moderate	Low	Low
7	Lopriore	Low	Low	Low	Low	Low	Low
8	Hack	Low	High	Moderate	High	Low	Moderate
9	Pruetz	Low	Low	Moderate	Low	Low	Low
10	Pettit	Low	Low	Moderate	Low	Low	Low
11	Springer	Low	Moderate	Low	Moderate	Low	Moderate
12	Eschbach	Low	Moderate	Low	Moderate	Low	Low
